# Quality Evaluation of Phellodendri Chinensis Cortex by Fingerprint–Chemical Pattern Recognition

**DOI:** 10.3390/molecules23092307

**Published:** 2018-09-10

**Authors:** Xuexiao Cao, Lili Sun, Di Li, Guangjiao You, Meng Wang, Xiaoliang Ren

**Affiliations:** 1 School of Chinese Materia Medica, Tianjin University of Traditional Chinese Medicine, Tianjin 300193, China; xuehen.3@163.com (X.C.); 18322050489@163.com (L.S.); 18222042511@163.com (D.L.); guangjiao_you@163.com (G.Y.); 2 Tianjin State Key Laboratory of Modern Chinese Medicine, Tianjin University of Traditional Chinese Medicine, Tianjin 300193, China

**Keywords:** Phellodendri Chinensis Cortex (PCC), Phellodendri Amurensis Cortex (PAC), fingerprint, chemical pattern recognition, quality evaluation

## Abstract

Phellodendri Chinensis Cortex (PCC) and Phellodendri Amurensis Cortex (PAC) are increasingly being used as traditional herbal medicines, but they are often mistaken for each other. In this study, the fingerprints of PCC from six different geographical sources were obtained by high-performance liquid chromatography, and multivariate chemometric methods were used for comprehensive analysis. Two unsupervised pattern recognition models (principal component analysis and hierarchical cluster analysis) and a supervised pattern recognition model (partial least squares discriminant analysis) were established on the basis of the chemical composition and physical traits of PCC and PAC. PCC and PAC were found to be distinguishable by these methods. The PCC category was divisible into two categories, one with more crude cork and a maximum thickness of ~1.5 mm, and the other with less net crude cork and a maximum thickness of 0.5 mm. According to the model established by partial least squares discriminant analysis (PLS-DA), the important chemical marker berberine hydrochloride was obtained and analyzed quantitatively. From these results combined with chemometric and content analyses, the preliminary classification standards for phellodendron were established as three grades: superior, first-order and mixed. Compared with the traditional identification methods of thin layer chromatography identification and microscopic identification, our method for quality evaluation is relatively simple. It provides a basis and reference for identification of PCC and enables establishment of grade standards. It also could be applied in quality control for compound preparations containing PCC.

## 1. Introduction

Phellodendri Chinensis Cortex (PCC) and Phellodendri Amurensis Cortex (PAC) are well-known traditional Chinese medicines (TCMs) known as Huangbai, from the dried bark of *Phellodendron chinense* Schneid, and Guanhuangbai, from the dried bark of *Phellodendron amurense* Rupr, respectively [1]. Currently, PCC is widely used for the treatment of diabetes, tumors, and other diseases [2,3]. It contains numerous chemical components, such as alkaloids and terpenoids, which confer multiple pharmacological activities [4,5,6]. Alkaloids, such as berberine and jatrorrhizine, have anticancer, antimicrobial, and neuroprotective activities [7,8,9], whereas limonoids, such as obacunone and obaculactone, have anticancer properties [10]. In addition, PCC and PAC also have anti-gout and anti-inflammatory effects and affect the immune system [11,12,13]. PCC and PAC were officially regarded as the same herb, ‘Huangbai’, in the Chinese Pharmacopoeia until the 2005 edition. These two herbs share similar clinical uses at the same dose of 3–12 g/day, although the high disparity in their berberine content, the major alkaloid in PAC (0.6%) and in PCC (3.0%), has given rise to doubts regarding the bioequivalence of the two drugs [1]. The difference between PCC and PAC had also attracted the attention of researchers. Sun et al. developed a multivariate analysis approach method to discriminate Cortex Phellodendri Amurensis and Cortex Phellodendri Chinensis [14]. Chen et al. distinguished Cortex Phellodendri Chinensis and Cortex Phellodendri Amurensis on a chemical and a biological basis [15]. According to the TCM theory, the therapeutic efficacy of herbal medicines derives from a combination of their bioactive components rather than from single components. Moreover, there are still many preparations on the market that contain mixtures of PCC and PAC. Therefore, it is important to develop a feasible method for evaluating quality and allowing for rapid identification of traditional herbal medicines.

So-called fingerprints reflect characteristic profiles of the samples; among these, chromatographic fingerprints are the most widely used tool to analyze natural samples [16]. However, the lack of overall analysis and comprehensive information on a large number of chemical constituents of TCM obtained by fingerprinting often results in a waste of data resources. Chemometrics [17,18] uses computer systems and corresponding programs to analyze the spectral and chromatographic data of medicinal extracts; this analysis uses statistical or mathematical methods to obtain useful information for analysis and identification and to establish relationships between the measured values of the chemical system and the state of the system. It can be used to solve common problems in determining fingerprints for natural samples, and it provides a variety of analysis and recognition patterns [19].

In this study, a chemometric strategy combining chemical pattern recognition with chromatographic fingerprints was developed to achieve standardization in TCM. Multiple pattern recognition models were established by hierarchical cluster analysis (HCA), principal component analysis (PCA) and partial least squares discriminant analysis (PLS-DA). The chemical markers were obtained by the PLS-DA model, which verified the reliability of the results, and by quantitative analysis of chemical markers with important pharmacological activities. The method enabled the classification and identification of PCC and PAC, and provides the first description of linkages among the appearance, texture and chemical composition of PCC. These results, combined with chemometrics and content analysis, allowed us to develop preliminary classification standards for phellodendron into three grades: superior, first-order, and mixed. This method is easy to perform and can be applied to analyze a large number of samples in short time. It provides a means of objective evaluation and identification for PCC. The proposed strategy provides a more comprehensive way to evaluate TCM quality.

## 2. Methods

### 2.1. Materials and Reagents

The 20 batches of PCC samples were collected from six different provinces (Sichuan, Shanxi, Hunan, Hubei, Yunnan and Guizhou) of China, and five batches of PAC samples were purchased from Heilongjiang, Hebei, and Liaoning in China. They were identified as the dried bark of *Phellodendron chinense* Schneid and the dried bark of *Phellodendron amurense* Rupr, respectively, by Professor Li Tianxiang of the Tianjin University of Traditional Chinese Medicine. The sample information is shown in Table 1. High performance liquid chromatography (HPLC)-grade methanol and acetonitrile were purchased from Sigma-Aldrich (St. Louis, MO, USA). Hydrochloric acid and HPLC-grade phosphoric acid was obtained from Tianjin Kemiou Chemical Reagent Co., Ltd. (Tianjin, China). Water used as a chromatographic mobile phase was purified with a Milli-Q system (Millipore, Bedford, MA, USA). Berberine hydrochloride (purity > 98%) was purchased from Shanghai Harmony Medical Technology Co., Ltd. (Shanghai, China).

### 2.2. Sample Preparation

PCC and PAC samples from various batches were collected, ground to a fine powder, and passed through a 50-mesh sieve. Then 0.2 g were weighed to the nearest 1 mg of each powder and added to methanol (10 mL, 0.1% hydrochloric acid made by diluting HCl with MeOH) and sonicated (150 W, 40 kHz) for 40 min, then make up weightlessness. Each sample was processed in parallel three times in the analysis.

A standard solution containing 0.50 mg of berberine hydrochloride per 1 mL was prepared by dissolving in methanol.

### 2.3. Chromatography Analysis

A Shimadzu HPLC system (SHIMADZU-LC-20-AT, Kyoto, Japan) equipped with a binary pump, an autosampler, a photodiode 99 array detector, and a temperature-controlled column oven was used to determine the fingerprint-chemical composition. Chromatographic separation was performed on an HPLC Symmetry^®^ C18 column (4.6 × 150 mm, 5.0 μm particle size; Waters, Milford, MA, USA), operated at 30 °C. The mobile phase was composed of acetonitrile (solvent A) and water containing 0.2% phosphoric acid (solvent B). A gradient elution program was used as follows: 0–20 min (5–10% A), 20–50 min (10–20% A), 50–60 min (20–25% A) and 60–75 min (20–100% A), with a mobile phase flow rate of 1 mL/min. Re-equilibration of the column with 5% A for ten minutes before each injection with a mobile phase flow rate of 1 mL/min. The detection wavelength was set at 254 nm with an injection volume of 5 μL. A photodiode array detector was used to collect chromatographic information from 200 to 400 nm. All solutions were filtered through a 0.22-μm nylon membrane by using syringe filters before injection into the HPLC.

A Waters Acquity UPLC system was used to access chemical information for chemical pattern recognition. Chromatographic separation was performed on an Acquity UPLC BEH shield RP18 column (100 mm × 2.1 mm, 1.7 μm particle size; Waters), operated at 30 °C. The mobile phase was the same as that described above. A gradient elution program was used as follows: 0–5 min (10–40%, A) and 5–10 min (40–100%, A), with a flow rate of 0.3 mL/min, and the detection wavelength was set from 210 to 400 nm with an injection volume of 3 μL.

### 2.4. HPLC Methodological Evaluation

Six successive injections of the same PCC sample solution were subjected to chromatography as described in Section 2.2. The relative retention time and relative peak area of each common peak were calculated by using berberine hydrochloride as the reference peak. The RSD for the relative peak retention time was <2.0% and the RSD for the relative peak area was <3.0%, indicating good precision.

Six samples of PCC were prepared in parallel from the same group of PCC samples according to the method in Section 2.2. The relative retention time and relative peak area of each common peak were calculated by using berberine hydrochloride as the reference peak. The RSD for the relative peak retention time was <1.6% and the RSD for the relative peak area was <2.67%, indicating good repeatability.

Samples of the same PCC solution, prepared according to Section 2.2, were analyzed at 0, 2, 4, 8, 12, and 24 h. The relative retention time and relative peak area of each common peak were calculated by using berberine hydrochloride as the reference peak. The RSD for the relative peak retention time was <2% and the RSD for the relative peak area was <3%, indicating that the sample was stable for 24 h.

### 2.5. Software

LabSolutions/LCsolution workstation data management software (2010 version, SHIMADZU, Kyoto, Japan) was used to collect chromatographic data on samples of PCC and PAC, including peak area, retention time and other information used to establish fingerprints. Waters Empower workstation data management software was used to determine chemical compositions for chemical pattern recognition. SOP of similarity evaluation system for chromatographic fingerprint of TCM 2004A software (Chinese Pharmacopoeia Commission, Beijing, China) was widely used for evaluating the fingerprint similarity of TCM in China [20]. Multivariate chemometric classification methods, including HCA, PCA, and PLS-DA, were applied to classify the new matrix data, determine the similarities and differences, and identify the marker components. SPSS 19.0 (IBM, San Diego, CA, USA) was used to build an HCA unsupervised pattern recognition model. SIMCA-P11.5 (Sartorius Scientific Instrument Co., Ltd., Beijing, China) was used to establish a PCA unsupervised pattern recognition model and a PLS-DA supervised pattern recognition model.

## 3. Results

### 3.1. Similarity Evaluation

**Figure 1:**
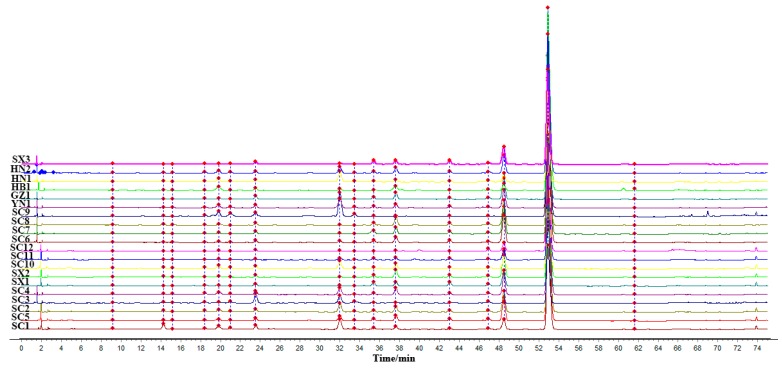
HPLC chromatographic fingerprints of PCC from six different geographical regions.

Chromatographic data from 20 PCC batches, collected as described in Section 2.3, were imported into the software SOP of Similarity evaluation system for chromatographic fingerprint of TCM 2004A. Using chromatogram of SC5 as a reference map, and multipoint correction, automatic matching and the median method to generate a control map, we generated the fingerprint superposition, as shown in Figure 1. The fingerprint similarity calculated for each sample was 0.981–1.000, thus indicating that the difference between PCC was smaller when the similarity was close to 1. The results are shown in Table 2.

### 3.2. HCA Modeling

**Figure 2:**
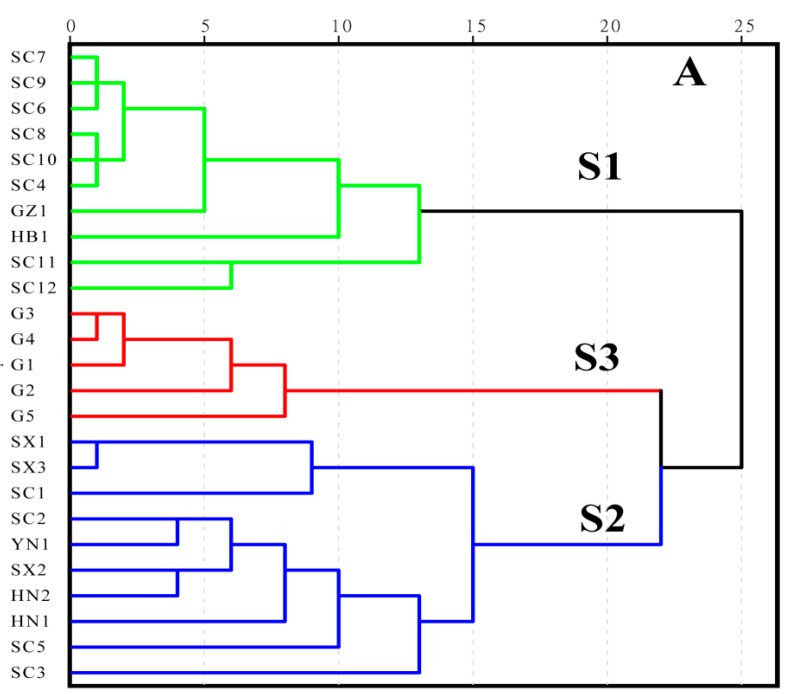
HCA of PCC and PAC (S1: PCC-more crude cork and a maximum thickness of ~1.5 mm; S2: PCC-less net crude cork and a maximum thickness of 0.5 mm; S3: PAC).

This clustering analysis method [21] is an unsupervised pattern recognition method based on a set of unclassified samples. From the dendrogram, we were able to easily and intuitively determine the similarities and differences of the tested samples [22]. According to the characteristics of the variables, the method classifies the degree of similarity among them. Our goal was to identify objective categories of the patterns for PCC and PAC. In this experiment, we used Waters Empower workstation data management software to access the peak area, retention time, and other related information. The obtained data matrix was imported into SPSS 19.0, and the HCA was carried out by using the average Pearson correlation (intragroup) linkage (Figure 2). When the distance scale was approximately 22, PCC and PAC were distinguishable, with S3 for PAC, and S1 and S2 for PCC. The PCC could be further divided into two categories, S1 and S2, on the basis of the appearance of the PCC samples, including shape, size, color, and texture. S1 was characterized by more crude cork and a maximum thickness of ~1.5 mm, whereas S2 was characterized by less net crude cork and a maximum thickness of 0.5 mm. The HCA model was able to distinguish the PCC and PAC on the basis of chemical composition to a certain extent, showing a significant relationship between the chemical composition and the sources/traits.

### 3.3. PCA Modeling

**Figure 3:**
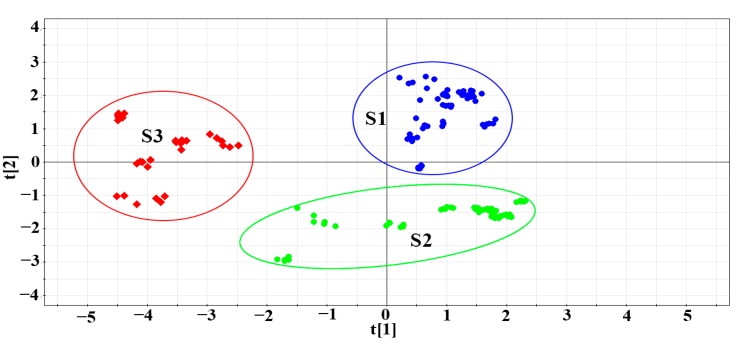
2D PCA score plot (PC1 versus PC2) of PCC and PAC samples, as listed in Table 1 (S1: PCC-more crude cork and a maximum thickness of ~1.5 mm; S2: PCC-less net crude cork and a maximum thickness of 0.5 mm; S3: PAC).

The PCA algorithm [23] is a linear method that converts raw data into new orthogonal variables (principal components, PC), which are combinations of the raw variables. The obtained data matrix was imported into SIMCA-P 11.5, and then PCA was used to describe the distribution of PCC and PAC. By using the principle of maximum variance, the multiple variables of the original data were fitted linearly to generate new low-dimensional variables to replace the original high-dimensional variables. Figure 3 shows the two-dimensional (2D) scatter plot of the main component score for classification. S3 was PAC, whereas S1 and S2 were PCC. This model validated the HCA results and provided the PC for identification of PCC and PAC on the basis of sources and traits. From the obtained eigenvalues, PC were selected, and the cumulative contribution rate R^2^X (cum) = 0.727 was used in subsequent analysis to reflect most of the fingerprint information. The cross-validation coefficient was 0.418, which indicated that the model was reasonable and had good analytical and predictive ability.

### 3.4. PLS-DA Modeling

**Figure 4:**
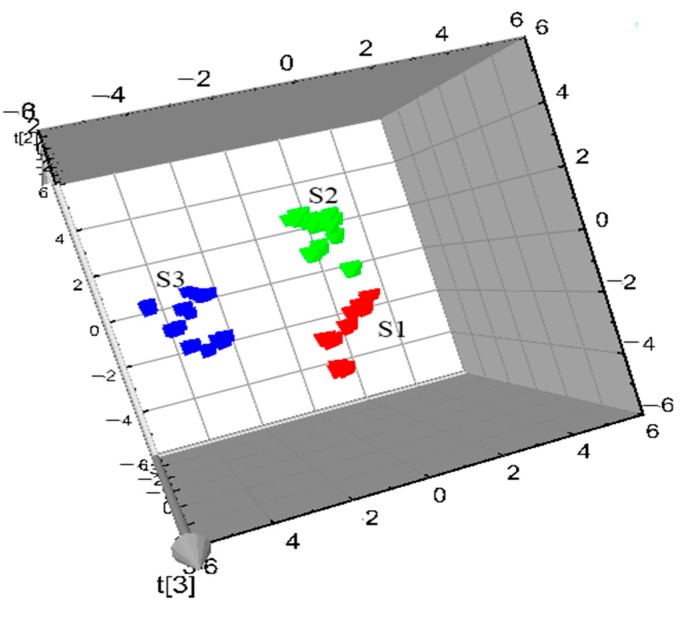
3D PLS-DA score plot (PC1 versus PC2) of PCC and PAC samples, as listed in Table 1 (S1: PCC-more crude cork and a maximum thickness of ~1.5 mm; S2: PCC-less net crude cork and a maximum thickness of 0.5 mm; S3: PAC).

PLS is a widely used stoichiometric method [24] that combines the advantages of multiple linear regressions with principal component regression, and has strong predictive ability in a relatively simple model. In this experiment, the data matrix obtained from the Waters Empower workstation were imported into SIMCA-P 11.5 software, the PLS-DA model was established, and chemical markers were obtained. The 3D score plots of PLS-DA are shown in Figure 4, in which the PCC and PAC were clearly distinguished. According to shape and size, the PCC could be divided into two categories: S1, with higher crude cork content and thickness, and S2, with lower net crude cork and thickness. The relevant information was reflected in the fingerprints, and this model provided more accurate identification. The thickness of crude cork may thus be used for classifying the quality PCC. The cross-validation Q^2^ (cum) in the PLS-DA model was 0.962. Feature value selection based on the cross-validation optimum number provided two PC with cumulative contribution rates R^2^X (cum) = 0.839 and R^2^Y (cum) = 0.963. This model had better accuracy compared to HCA of identification of PCC and PAC according to different sources/traits.

**Figure 5:**
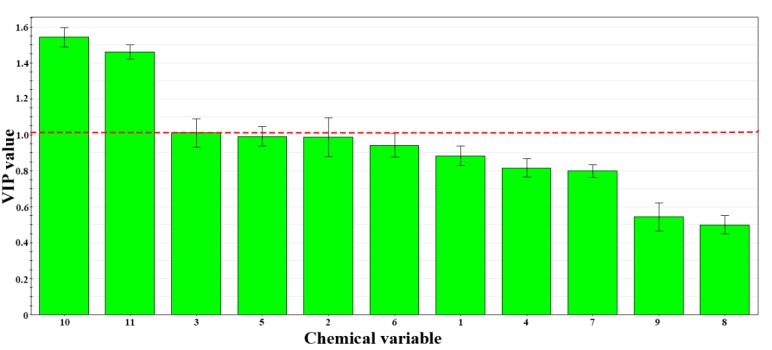
VIP plot of PLS-DA.

To determine the importance of each variable for discrimination and the obtained chemical markers, a variable importance for the project (VIP) [25] plot (Figure 5) was generated. Many chemical variables had a VIP value greater than 1 and therefore played an important role in discrimination. Among them, variables 10 and 11 had the largest VIP values and were the main components of the PLS-DA model that was used to fit the data and had good predictive value when applied to new data. Peak 10 was berberine hydrochloride, as determined by standard product identification, and peak 11 is an unknown component in PAC.

**Figure 6:**
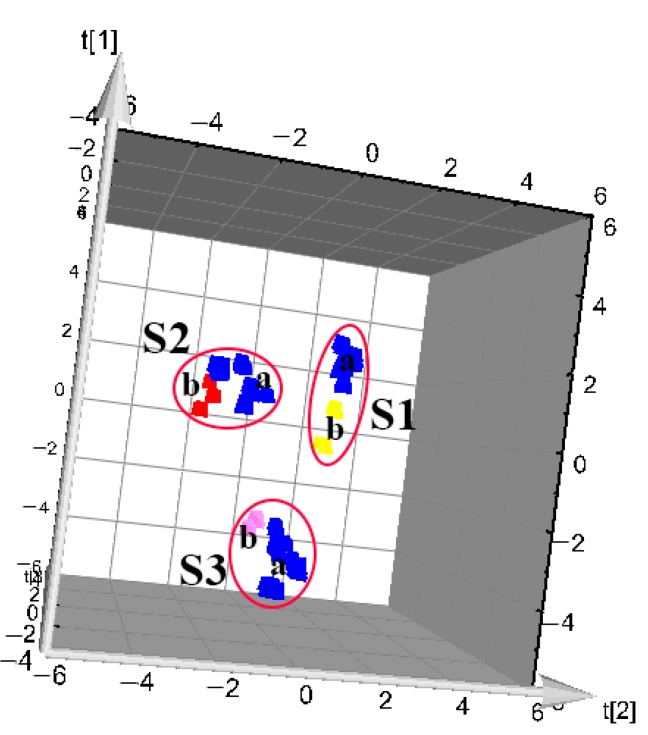
Unknown sample prediction by PLS-DA. (S1: PCC-more crude cork and a maximum thickness of ~1.5 mm; S2: PCC-less net crude cork and a maximum thickness of 0.5 mm; S3: PAC.) (**a**) Training set; (**b**) prediction set.

In order to further verify the accuracy of the model, two batches of PCC and PAC from Sichuan were collected respective. The unknown sample prediction by PLS-DA was shown in Figure 6 (S1: PCC-more crude cork and a maximum thickness of ~1.5 mm; S2: PCC-less net crude cork and a maximum thickness of 0.5 mm; S3: PAC). In the figure, (**a**) is the training set, which was the classification model established by using known samples, and (**b**) is the prediction set, which included the unknown samples. All predicted samples were correctly identified. The results shown that the model could be used for analysis and prediction of PCC and PAC: the model fitting data was good and could predict the new data.

### 3.5. Determination of Berberine Hydrochloride

To accurately measure the amount of the reference substance berberine hydrochloride, a series of reference solutions at different concentrations was prepared by dilution. The peak areas were measured under a wavelength of 265 nm. The following regression equation of berberine hydrochloride was obtained: *Y* = 2E + 07*X* − 40531, with *Y* as the peak area and *X* (mg·mL^−1^) as the concentration. The correlation coefficient (*r*) was 0.9998, and the linear range was 0.015 to 0.6 mg·mL^−1^. The average recovery (*n* = 6) of berberine hydrochloride was 104.9%, and the RSD was 2.58%, which showed the accuracy was good. The content of berberine hydrochloride in 20 batches of PCC is shown in Table 3.

## 4. Discussion and Conclusions 

TCM have diverse and complex chemical compositions. Therefore, simply evaluating of one or several components in traditional herbal medicines cannot reflect their quality. TCM quality evaluation should combine appearance (such as shape, color, and taste) with effective components and integral components. In study, quality evaluation of PCC from two aspects: berberine hydrochloride content and appearance characters, moreover distinguished counterfeit PAC and PCC clearly. The results of chemometrics suggested that the main factor influencing the PCC quality was crude cork thickness, which was mainly related to the number of years of growth. An important chemical marker affecting the quality of PCC was screened by establishing a PLS-DA model: berberine hydrochloride. It was reported in the literature [26] that the thickness of the crude cork was related to the growth period, and the content of the active ingredient of the crude cork was low, which was consistent with the traditional method of removed the crude cork of traditional Chinese medicine. The results of the content determination showed that the thickness of the crude cork was higher due to the longer growth period, and the content of berberine hydrochloride was also higher. Quantitative analysis of berberine hydrochloride revealed a large difference in content in PCC. Therefore, superior quality PCC should have higher berberine hydrochloride content and high net crude cork. This study preliminary classified PCC from the content of berberine hydrochloride and the thickness of the crude cork as three grades: (1) Superior grade: high net crude cork, berberine hydrochloride content at least 6.50%; (2) first-order: less net crude cork and a maximum, thickness of 0.5 mm, berberine hydrochloride content at least 5.00%; (3) mixed: low net crude cork, berberine hydrochloride content at least 3.00%.

The fingerprint–chemical pattern recognition method applied in this study provides a comprehensive analysis of the overall composition of PCC. Results obtained by HCA, PCA and PLS-DA suggested that the counterfeit PAC and PCC were substantially different, and thus mixing them is not advisable. A new method based on fingerprint-chemical pattern recognition for quality control and identification of PCC was thus developed. This method may also provide new applications for the research on TCM and natural samples.

## Figures and Tables

**Table 1:** Description of PCC and PAC samples.

Sample Name	Traits	Crude Cork Thickness	Origin	Source
SC11-SC12-SC13	Strip; Thickness 1–3 mm	0.1–0.3 mm	Sichuan (SC)	PCC
SC21-SC22-SC23	Strip; Thickness 1–2 mm	-		
SC31-SC32-SC33	Strip; Thickness 1–2 mm	-		
SC41-SC42-SC43	Strip; Thickness 1–3 mm	0.5–1.3 mm		
SC51-SC52-SC53	Strip; Thickness 1–3 mm	-		
SC61-SC62-SC63	Strip; Thickness 1–3 mm	0.6–1.3 mm		
SC71-SC72-SC73	Strip; Thickness 1–4 mm	0.6–1.5 mm		
SC81-SC82-SC83	Strip; Thickness 2–4 mm	0.6–1.5 mm		
SC91-SC92-SC93	Strip; Thickness 2–4 mm	0.8–1.5 mm		
SC101-SC102-SC103	Strip; Thickness 1–3 mm	0.6–1.5 mm		
SC111-SC112-SC113	Slice; Thickness 1–3 mm	0.7–1.2 mm		
SC121-SC122-SC123	Slice; Thickness 1–3 mm	0.6–1.3 mm		
SX11-SX12-SX13	Strip; Thickness 3–8 mm	-	Shanxi (SX)	PCC
SX21-SX22-SX23	Strip; Thickness 1–3 mm	0.1–0.3 mm		
SX31-SX32-SX33	Strip; Thickness 3–6 mm	0.1–0.5 mm		
HN11-HN12-HN13	Strip; Thickness 1–2 mm	-	Hunan (HN)	PCC
HN21-HN22-HN23	Strip; Thickness 1–3 mm	-	Hubei (HB)	PCC
HB11-HB12-HB13	Strip; Thickness 1–4 mm	0.6–1.2 mm		
GZ11-GZ12-GZ13	Slice; Thickness 3–6 mm	0.8–1.5 mm	Guizhou (GZ)	PCC
YN31-YN32-YN33	Strip; Thickness 3–5 mm	0.1–0.5 mm	Yunnan (YN)	PCC
G11-G12-G13-G21-G22-G23	-	-	Liaoning, Heilongjiang, Hebei. (G)	PAC
G31-G32-G33-G41-G42-G43				
G51-G52-G53				

**Table 2:** Similarity evaluation of 20 PCC batches.

Batches	Similarity	Batches	Similarity
SC1	0.998	SC11	0.999
SC2	1.000	SC12	0.998
SC3	0.981	SX1	0.996
SC4	1.000	SX2	0.997
SC5	1.000	SX3	0.997
SC6	0.998	HN1	0.997
SC7	0.998	HN2	1.000
SC8	0.997	HB1	0.996
SC9	0.988	GZ1	0.998
SC10	1.000	YN1	1.000

**Table 3:** The content of berberine hydrochloride in 20 PCC batches.

Batches	Berberine Hydrochloride (%)	Batches	Berberine Hydrochloride (%)
SC1	4.78	SC11	4.59
SC2	6.45	SC12	4.28
SC3	2.13	YN1	6.20
SC4	6.12	GZ1	5.07
SC5	6.15	HB1	7.68
SC6	6.03	HN1	4.10
SC7	6.54	HN2	5.51
SC8	5.64	SX1	3.25
SC9	6.45	SX2	5.63
SC10	4.65	SX3	3.65

## Abbreviations

HPLC	High-performance liquid chromatography
HCA	Hierarchical cluster analysis
PCA	Principal component analysis
PLS-DA	Partial least squares discriminant analysis
PCC	Phellodendri Chinensis Cortex
PAC	Phellodendri amurensis cortex
TCM	Traditional Chinese medicine
VIP	Variable importance for the project
